# Regulation of AU-Rich Element RNA Binding Proteins by Phosphorylation and the Prolyl Isomerase Pin1

**DOI:** 10.3390/biom5020412

**Published:** 2015-04-14

**Authors:** Zhong-Jian Shen, James S. Malter

**Affiliations:** Department of Pathology, University of Texas Southwestern Medical Center, Dallas, TX 75390-8548, USA

**Keywords:** AU-rich element, phosphorylation, prolyl isomerase Pin1, RNA-binding protein, signaling, gene regulation, mRNA turnover, kinase

## Abstract

The accumulation of 3' untranslated region (3'-UTR), AU-rich element (ARE) containing mRNAs, are predominantly controlled at the post-transcriptional level. Regulation appears to rely on a variable and dynamic interaction between mRNA target and ARE-specific binding proteins (AUBPs). The AUBP-ARE mRNA recognition is directed by multiple intracellular signals that are predominantly targeted at the AUBPs. These include (but are unlikely limited to) methylation, acetylation, phosphorylation, ubiquitination and isomerization. These regulatory events ultimately affect ARE mRNA location, abundance, translation and stability. In this review, we describe recent advances in our understanding of phosphorylation and its impact on conformation of the AUBPs, interaction with ARE mRNAs and highlight the role of Pin1 mediated prolyl *cis-trans* isomerization in these biological process.

## 1. Introduction

The cytoplasmic level of all mRNAs is dictated by a complex interplay between the rates of gene transcription and mRNA decay. mRNA decay allows cells to eliminate aberrant/mutant messages (nonsense-mediated) as well as modulate mRNA pools and abundance to rapidly adapt to an ever-changing environment [1,2]. The downstream consequence is a rapid (<1–2 h) cessation or augmentation of protein synthesis, irrespective of the transcription rate that may remain unchanged. As transcription may take hours or even days to substantively change, the modulation of mRNA stability, especially in mammalian cells, is one of the most powerful and rapid means to alter protein expression and cellular homeostasis.

In steady state mammalian cells, mRNAs show a wide variety of decay rates. Protein with critical cellular functions such as cytokines, oncogenes, kinases, phosphatases and cell-surface receptors are often, but not exclusively coded by short-lived mRNAs that are unstable in resting cells [1,2]. Along with protein turnover, rapid mRNA decay prevents protein expression and its biological consequences. Not surprisingly, this process is often deranged in tumor cells and likely facilitates their growth. The determinants of mRNA decay (along with mRNA localization) are typically embedded into the 3'-UTR, although 5'-UTR elements have also been described. The best characterized of these are the so-called adenosine-uridine (AU)-rich elements often found in 3'-UTR of early response genes (cytokines, lymphokines and proto-oncogenes). Bioinformatics analysis have suggested that, in humans, AREs occur in up to 5%~8% of all transcripts [3,4] although only 10%~15% of all ARE mRNAs have half-lives below 2 h [5]. These data suggest that other features may mitigate the destabilizing function of AREs, including their location relative to the stop codon or poly-A tail, the number of reiterations and the spacing between AREs as well as the adjacent, non-ARE sequences. Such sequence variation may alter secondary or tertiary mRNA structure, partially or fully accounting for the observed phenotypes.

Trans-acting factors (e.g., RNA-binding proteins and microRNAs), that bind directly or indirectly to the AREs [6] are essential components for regulated decay. Such binding proteins (ARE-BPs or AUBPs) participate broadly in ARE mRNA metabolism including their nuclear splicing, defect surveillance and cytoplasmic transport, as well as their cytoplasmic stability, translation and subcellular localization. Proteins can be loaded onto pre- or mature ARE mRNAs in both the nucleus and the cytoplasm. The first cytoplasmic AUBP was identified using RNA mobility shift assays with leukemia cell lysates and *in vitro* transcribed, radiolabeled ARE RNA fragments derived from GM-CSF mRNA [7]. AUF1 was the first AUBP to be cloned and approximately 20 additional AUBPs [8,9,10] have since been identified suggesting a class of proteins with a broad range of function and possible redundancy. It is worth noting that AUBPs recognize their targets through a combination of primary sequence and secondary, tertiary or potentially quaternary conformation. The latter sets AUBPs (and other RNA-binding proteins) apart from DNA binding proteins. Given this complexity, it is not surprising that relatively little is known about how AUBPs identify and interact with their targets and what signaling cascades affect their function.

AUBPs bind to AREs via a variety of domains including the so-called RNA-recognition motif (RRM), CCCH tandem zinc finger, and the K-homology domain (KH) [9]. A single protein may contain multiple motifs implying a capacity for simultaneous interactions with multiple targets or multiple sites within a single target. Perhaps, not surprisingly, AUBPs can accelerate (e.g., AUF1, TTP, and KSRP), or attenuate (e.g., HuR) ARE mRNA decay. Multi-isoform AUBPs such as AUF1 have been shown to both stabilize and destabilize target mRNAs [9], consistent with unique isoform functionality.

The breadth of AUBP-mediated regulation is substantial. For example, a genome-wide analysis identified ~250 mRNAs that were stabilized in TTP^−/−^ MEFs [11]. These results demonstrate that an individual AUBP can control the decay of many ARE mRNAs simultaneously and imply that despite their redundancy, that specific AUBPs cannot be complemented by the function of other AUBPs [12]. While 250 mRNAs is substantial, it represents far less than 5% of the predicted number of ARE mRNAs in a mammalian cell. Thus, these data also suggest there is likely a subset of ARE targets that are selective ligands of individual AUBPs. This is consistent with observations showing that some AUBPs have modest effects on mRNA decay but instead modulate the translation of mRNA targets. For example, TIA-1 and TIAR that bind the ARE in TNF-α mRNA inhibit translation without affecting mRNA decay kinetics. Mechanistically, these AUBPs relocalize target mRNAs from polysomes to untranslatable, mRNP stress granules [13].

The expression of different AUBPs varies depending on cell/tissue types and external stimuli. However, it is clear that multiple AUBPs coexist in cells and that numerous target mRNAs can interact with and presumably be regulated by multiple AUBPs. Thus, a critical question remains as to what determines the recognition between an AUBP and specific target ARE mRNA. As the interaction appears to be extremely plastic and dynamic, signal transduction events that trigger AUBP post-translational modification(s) seem likely to alter the affinity or localization (among other options) of preexisting AUBPs [14,15]. Indeed, AUBPs can be methylated [16], phosphorylated [17], glycosylated [18], and ubiquitinated [19]. Of these, protein-kinase-triggered phosphorylation has been implicated in directing the binding of AUBPs to protein cofactors (e.g., chaperones), mRNA targets and the ubiquitin-proteasome system [14,15,17].

Pin1, a *cis-trans* prolyl isomerase (PPIase), was cloned from a human cDNA library and found to be essential for cell-cycle progression [20]. Pin1 is highly conserved from yeast to humans and related to the *cis-trans* isomerases of the cyclophilin and FKBP families. While the latter proteins can isomerize X-Pro peptide bonds (where X is any amino acid), Pin1 is the only known mammalian isomerase with rigid specificity for Ser-Pro or Thr-Pro peptide bonds. Isomerization is bidirectional with *cis* to *trans* or *trans* to *cis* conversions but occurs approximately 1000 fold faster when the N-terminal Ser or Thr has been phosphorylated [21,22,23]. Structurally, Pin1 is bipartite with a 40 amino acid N-terminal, WW domain and a C-terminal isomerase domain [24,25]. The WW domain binds to pSer/pThr-Pro motifs while the catalytic domain is responsible for substrate isomerization. If Ser or Thr is dephosphorylated post-isomerization, the large difference in activity towards unphosphorylated substrates prevents further isomerization and essentially locks the new conformation in place. As *trans* pSer/pThr-Pro bonds often show enhanced phosphatase accessibility, this is likely a common event.

Pin1 mediated prolyl *cis-trans* isomerization has profound effects on target protein folding, altering subsequent protein-protein and protein-nucleic acid interactions, protein stability and subcellular localization thereby altering a variety of cellular processes including cell cycle progression, apoptosis, innate and acquired immunity, and gene regulation [21,22,26]. Recent studies have shown that Pin1 regulates cytokine gene expression and immune responses in several disorders (asthma, organ rejection, anti-viral immunity) [22].

In this review, we will discuss the post-translational modification of AUBPs by phosphorylation and potential role of Pin1 isomerization in the regulation of AUBPs conformational remodeling, AUBPs-RNA interaction and ARE mRNA turnover in mammals. Finally, we will address several physiological cytokines and immune disorders associated with the function of Pin1.

**Table 1:** AUBP phosphorylation, potential Pin1 binding sites and target mRNAs.

AUBP	Phosphorylation site *	Kinase	Interaction w/Pin1	mRNA stability affected by AUBP	mRNA stability/expression affected by Pin1
AUF1	**Ser83**, Ser87, Thr91	CK1, GSK3β, PKA [27,28,29]	Yes [30,31]	c-myc, c-fos, Cyclin D1, GM-CSF, iNOS, IL-1β, IL-2, IL-3, IL-6, IL-10, p21, PTH, TNF-α [32,33,34,35]	Cyclin D1, GM-CSF, IL-1β, IL-2, IL-6, PTH, TNF-α [30,36,37,38,39,40,41]
BRF1	Ser54, Ser92, Ser203	AKT, ERK2 [42,43,44]	N/D **	GM-CSF, IL-3, TNF-α [8]	GM-CSF, TNF-α [30,38,40]
DAZAP1	**Thr269**, **Thr315**	ERK2 [45,46]	N/D	Regulates RNA splicing and translation [45,46,47]	
hnRNP C	N/D	N/D	No [30,31,36]	APP, GM-CSF, TGF-β, Urokinase receptor [30,36,48,49,50]	GM-CSF, TGF-β [30,36,38]
HuR	Ser88, Ser100, Thr118, Ser158, **Tyr200, Ser202**, **Ser221**, Ser242, Ser318	AMPK, MAPKs, CDK1, CHK2, JAK3, PKCs [51,52,53,54,55,56,57,58,59,60,61,62,63]	Yes [30,31,36]	AFT-2, C/EBP-β, Cyclin A/B1/D1, Cox-2, cPLA2α, CXCL8, CXCL1/5, c-fos, Dll1, DNMT3B, GATA3, GM-CSF, iNOS, IL-3, IL-8, MyoD, Myogenin, Musashi1, NPM, p21, PEPCK, RGS4, SIRT1, SMN, Survivin, TNF-α, VEGF, VHL, XIAP [8,50,56,64,65,66,67,68,69,70,71,72,73,74,75,76,77,78]	Cyclin D1, Cox-2, GM-CSF, iNOS, IL-8, TNF-α, VEGF [30,38,40,79,80]
KSRP	Ser193, **Thr692**	AKT, p38 MAPK [81,82]	Yes [37]	β-catenin, c-fos, c-jun, GAP43, IL-2, iNOS, MyoD, Myogenin, p21, TNF-α [8,64,81,82,83]	IL-2, iNOS, β-catenin, TNF-α [40,84,85,86,87]
La (SSB)	Thr301, Ser366, Thr389	AKT, CK2 [88,89,90,91]	N/D	Regulates RNA translation [88,90,91]	
NF90	Ser482, Ser647	AKT, PKC-β [92,93,94]	N/D	Cyclin E1, IL-2, MKP-1 [95,96,97]	IL-2 [84]
Nucleolin	Thr641, Thr707	CDK1,CDC2,CK2,GSK3β,PI3K [98,99,100,101,102]	No [30]	Bcl-xL, β-globin, CD154, GM-CSF, Gadd45a, Gastrin, HIF1α, IL-2, p53 [18,102,103,104,105,106,107,108,109,110]	IL-2, GM-CSF [30,38,84]
SLBP	Ser60, **Thr62**, **Thr171**	N/D [111,112,113]	Yes [26,31]	Histone [26]	Histone [26]
TIA-1/R	N/D	FASTK [114,115,116]	No [30,31,36]	Regulates RNA translation [13]	
TTP	Ser52, Ser66, **Ser88**, Thr92, **Ser93**, Ser169, Ser178, **Ser186**, **Ser197**, Ser218, **Ser220**, **Ser228**, **Ser245**, Thr250, Ser276, Ser296	AKT, GSK3β, MK2, MAPKs, PKA, PKC [117,118,119,120,121,122,123,124,125]	N/D	Bdp1, Claudin-1, Cyclin D1, c-fos, Cox-2, GM-CSF, IL-1α, IL-2, IL-3, IL-6, IL-8, IL-10, IL-23, ler3, IFN-γ, iNOS, LIF, Mllt11, c-myc, Pim3, PLK3, PHLDA1, PAI-2, Pitx2, Rusc2, TNF-α, VEGF [32,126,127]	Cox-2, GM-CSF, IL-2, IL-8, IFN-γ, iNOS, TNF-α, VEGF [30,38,40,79,84,85]
YB1	Ser316	AKT, ERK2, GSK3β,JNK [103,128,129,130]	No [30]	IL-2 [32,103,128]	IL-2 [84]

* Phosphorylation residues highlighted in bold are the potential Pin1 binding sites (Pro-Ser/Thr). **N/D: not determined.

## 2. Post-translational Regulation of AUBPs by Phosphorylation

Kinases involved in AUBP phosphorylation and ARE mRNA stability include the proline directed kinases (MAPKs, CDKs and FA/GSK3) and others (AMPK, PKCs, PKA, CKs, and PI3K/AKT) (Table 1). The MAPKs and PI3K/AKT are the most studied signaling pathways regulating ARE-mediated post-transcriptional events [131] (Table 1). How the triggering of these signaling pathways results in modulation of ARE mRNA decay remains poorly understood. These kinases are associated with phosphorylation of a range of AUBPs at serine or threonine residues, including TTP (Ser52 and Ser178) [117], DAZAP1 (Thr269 and Thr315) [45], La (Ser366) [88], HuR (Ser221 and Ser318) [51], KSRP (S193 and T692) [132], AUF1 (Ser83 and Ser87) [133], BRF1 (S54, S92, S203 and T270) [42] and NF90 (Ser647) [92] (see Table 1 for details). Despite this growing list of kinase sites, the functional significance of these events remains murky. Nonetheless, as blockade of signal transduction or specific kinases clearly alters ARE mRNA decay, these phosphorylation events must be critically important and likely relevant to many diseases such as pathologic inflammation and cancer [131]. Here, we will selectively focus on the phosphorylation of a subset of AUBPs that has been most extensively studied and are potential Pin1 targets.

TTP is a destabilizing AUBP that can be phosphorylated in murine cells by MK2 (substrate of p38 MAPK) at two serine residues [117]. Phosphorylation is required for TTP to bind the 14-3-3 adaptor protein, attenuating its ARE mRNA destabilizing activity [118,134]. Serine phosphorylation is also associated with the nuclear export of TTP in response to mitogens or serum [119,135]. TTP deficient mice stabilize TNF-α mRNA leading to massive overproduction of TNF-α [119]. Not surprisingly, the mice suffer from severe spontaneous inflammation and autoimmunity manifested as arthritis and dermatitis. Consistent with a regulatory role, MK2^−/−^ knockout mice show low TNF-α levels while double MK2^−/−^:TTP^−/−^ mice express high levels of TNF, reminiscent of TTP^−/−^ mice [120]. These data suggest that TTP phosphorylation (Ser52 and Ser178) by MK2 and subsequent interaction with adaptor molecules reduces ARE mRNA affinity and/or impairs the recruitment of the mRNA decay machinery (e.g., exosome) to TNF transcripts. As PKC-δ and IKKβ-induced phosphorylation of TTP can also interfere with the mRNA binding and destabilizing actions of TTP [136], it is likely that there are redundant kinase signals that regulate TTP. MK2 can be counterbalanced by the protein phosphatase PP2A, which directly competes with 14-3-3 protein for binding to TTP [137]. Dephosphorylation of TTP by PP2A activated ARE mRNA decay. This function of PP2A may be of particular importance during immune response when cytokine levels change and the coding mRNAs are rapidly metabolized. Well-established target mRNAs for TTP binding include TNF-α, GM-CSF, IL-2, IL-3, c-fos, COX-2 and VEGF. Transcriptome analysis using RNA from TTP wild-type and knockout cells identified additional target mRNAs [11,12] (Table 1). In summary, TTP may constitute an attractive AUBP for novel anti-inflammatory therapeutic concepts.

AUF1 (hnRNP D) appears to be unique as an AUBP with both stabilizing and destabilizing activity. It can destabilize (c-myc, c-fos, GM-CSF, IL-3, p21, cyclin D1, and iNOS) or stabilize (c-myc, c-fos, GM-CSF, IL-1β, TNF-α, PTH) ARE mRNAs (Table 1). Alternative splicing of AUF1 transcripts yields four different protein isoforms p37, p40, p42 and p45. Each exhibits unique biochemical characteristics that mediate distinctive RNA-phenotypes. Thus, the regulatory control by AUF1 isoforms appears to be complex than other AUBPs and is further expanded by the potential for AUF1 proteins to form heterodimers [138] and by post-translational modifications of specific isoforms. Indeed, each isoform shows a different affinity for ARE containing mRNAs (p37 > p42 > p45 > p40) and can be expressed differentially in cell-specific fashion [139,140]. The opposing effects of AUF1 on mRNA stability may result from the relative levels of each isoform in a given cell type or in response to a specific stimulus. The p37 isoform has been shown to interact with the exosome and to exhibit the greatest destabilizing activity toward ARE-containing mRNAs but similarly detailed analysis of the properties of other isoforms has not been reported. All isoforms can undergo post-translational modifications such as methylation [141], ubiquitination [142] and phosphorylation, all of which affects mRNA binding affinity, intracellular trafficking and protein binding affinity to other associated factors [143].

The first phosphorelationsites in p40 AUF1 were identified on Ser83 and Ser87 in polysome-associated protein [27,28]. These serines are encoded by exon 2, which are absent in p37 and p42 AUF1. In THP-1 monocytic leukemia cells, stimulation with phorbol ester induced dephosphorylation of these residues concomitant with stabilization of several candidate AUF1-binding mRNAs (IL-1β and TNF-α) [28,133]. In contrast, p40 AUF1 phosphorylation at these sites destabilized target mRNAs. Because p37 AUF1 lacks these residues, activation of signaling pathways could selectively activate or inactivate p40. In the absence of p40, p37 function may be unopposed. p40 AUF1 can be phosphorylated *in vitro* on Ser87 and Ser83 by protein kinase A (PKA) and glycogen synthase kinase 3 beta (GSK3β), respectively [144] (Table 1). The *in vivo* action of these kinases on AUF1 has not been confirmed et but Ser83 phosphorylation may require prior Ser87 phosphorylation. Phospho-Base predicted seven additional phosphorylation sites, but to date, only CK1 site at Thr91 has been investigated [29]. The fact that all three sites mapped to exon 2 suggests that p45 AUF1 might also be similarly phosphorylated although this hypothesis has not been confirmed. Furthermore, cellular radiolabeling and two-dimensional Western analyses indicated that other AUF1 isoforms can also be modified on Ser, Thr and Tyr residues [145], Collectively, these observations imply diverse regulation of AUF1 isoforms. In cancer cells, the fusion oncokinase NPM-ALK bound to, and hyperphosphorylated all AUF1 isoforms [145]. Mass spectrometric analysis revealed p45 AUF1 bound to NPM-ALK, and both proteins colocalized within cytoplasmic granules. Under these conditions, c-myc and cyclin ARE mRNAs were stabilized with potential oncogenesis [145].

HuR promotes ARE mRNA stability and translation through high affinity binding to the ARE. Overexpression of HuR substantially increases the half-life of many short-lived mRNAs, including those coding ATF-2, Cox-2, XIAP, c-fos, p21, iNOS, GM-CSF, VEGF, TNF-α, IL-3, IL-8, IL-13, COX-2, and cyclins (Table 1). HuR-dependent, mRNA stabilization seems closely linked to its subcellular localization and phospho-status. While HuR is predominantly nuclear, it can translocate to cytoplasm upon cellular activation [52,146,147]. Nuclear export of HuR was stimulated in response to various stimuli via MAPK [148,149], AMPK [150], Cdk1 [151] and PKC family [152]. Under these conditions, HuR was phosphorylated at many sites (Ser88, Ser100, Thr118, Ser158, Ser202, Ser221, and Ser318) [51,53,54,55,153,154] through the action of PKC-α/-delta, Cdk1, Chk2 and p38 MAPK. HuR contains no canonical phosphorylation sites for Erk or JNK MAPK, suggesting alterations in HuR translocation and ARE mRNA binding affinity are mediated primarily by PKCs, Cdk1 and Chk2 [144]. One study [56] showed that phosphorylation at Tyr200 by JAK3 attenuated HuR localization to stress granules (SG) and reduced HuR interactions with targets SIRT1 and VHL mRNAs. Under these conditions, both mRNAs decayed more quickly. To date, the precise mechanism and overall impact of kinases in HuR-dependent mRNA stabilization remain incompletely understood.

**Figure 1:**
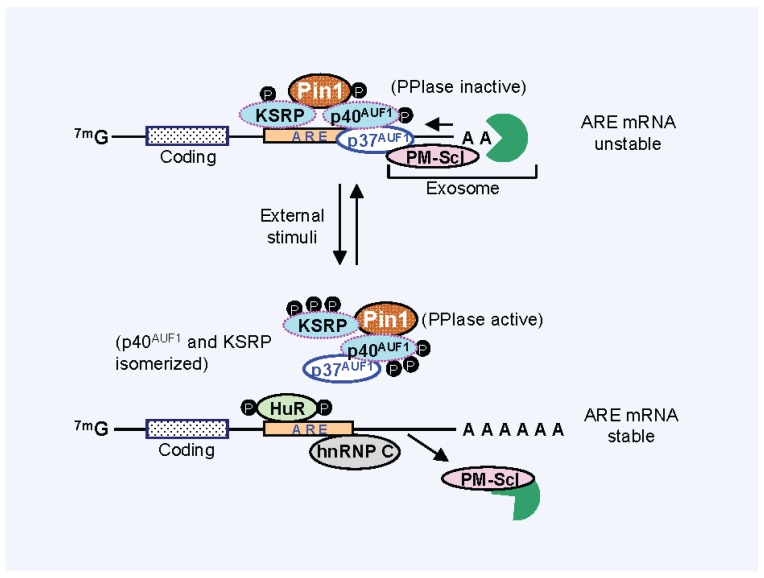
Regulation of ARE mRNA turnover by prolyl *cis-trans* isomerase Pin1. In response to external stimuli, AUBPs rapidly undergo post-tranlational modification by phosphorylation and dephosphorylation. Pin1 binds phosphorylated AUBPs (p40 AUF1 and KSRP) at pSer/pThr-Pro motifs and, upon activation by dephosphorylation, isomerizes the AUBPs. This cause reconstitution and remodeling of AUBPs-mRNA-exosome complex resulting in changes in the interaction between AUBPs and mRNA as well as mRNA and the exosome [30,36,37,38,64,81,82,155].

The p38-dependent regulation of KSRP plays an important role in the turnover of myogenic transcripts of p21, myogenin and MyoD [81]. Other mRNAs targeted by KSRP include c-fos, iNOS and TNF-α (Table 1). Phosphorylation of KSRP reduced its binding to ARE-containing transcripts, thus reducing their rapid decay [81]. AKT-mediated phosphorylation at a unique serine residue within the N-terminal, KH domain (KH) of KSRP inactivated the destabilizing activity of KSRP, possibly by enhancing interactions with 14-3-3 and blocking association with the exosome [155]. These regulatory pathways are thus similar to that seen with AUF1 (Figure 1).

## 3. Pin1 Is Associated with AUBPs and Regulates Cytokine mRNA Stability

As discussed above, the regulation of ARE mRNA decay depends on the regulated phosphorylation of a variety of AUBPs. Under normal conditions, post-transcriptional mechanisms rapidly and substantially alter the levels of ARE mRNAs. As most pro-inflammatory cytokines are coded by ARE-containing mRNAs, this process is essential to ramp up cytokine expression after infection or injury as well as quench expression for a return to normal homeostasis. While intracellular phosphorylation clearly altered the assembly and ARE binding affinity of p40 AUF1 [27,28,143], we were unable to reproduce these findings with *in vitro* synthesized GM-CSF mRNA and mutant AUF1 isoforms [156]. These results suggested that additional steps/regulators were involved in modulating AUF1 function after phosphorylation. Co-immunoprecipitation and gene knockout studies have shown that Pin1 interacts with multiple AUBPs (AUF1, HuR, and KSRP,) and RNA-binding protein (SLBP) in tumor and immune cells [30,31,36] (Table 1) although the interaction may depend on cell type and environmental stimuli. All these molecules have 1–3 canonical Pin1 binding sites (Ser/Thr-Pro) (highlighted in bold in Table 1) that are phosphorylated by various kinases. DAZAP1 and TTP have several phosphorylation Pin1 sites and their interaction with Pin1 has not been determined. Functionally, Pin1 modulated the expression of a number of cytokines/growth factors (GM-CSF, TGF-β, IL-4, IFN-γ, IL-1β, CXCL-10, PAI-1, FGF-1, TSLP, CTGF, IL-2, and IL-5) in various cell types (leukocytes, T cells and mesenchymal cells) and tissues [30,36,84,157,158].

The same signaling cascades that mediate AUBP phosphorylation appear to act on Pin1 to modulate its activity [30,36]. While Pin1 levels and nuclear localization show gradual changes during the cell cycle, Pin1 isomerase activity can be rapidly (seconds to minutes) modulated by cytokine-driven signaling [30,36,84,157,158], presumably coincident with the phosphorylation of target sites. Activity can be measured in cell lysates using a pentapeptide substrate, permitting analysis of Pin1 regulation. Importantly, primary eosinophils and T cells obtained from patients with active asthma showed significantly elevated Pin1 isomerase activity that mirrored data from *in vitro* activated cells obtained from healthy donors [84,156]. Therefore, the modulation of Pin1 activity occurs in human disease and can be modeled through *in vitro* agonists.

In the context of AUBPs, Pin1 constitutively interacted with the AUF1 complex in eosinophil cytoplasm [30] (Figure 1). Cell activation either *in vivo* or *in vitro*, triggered Pin1 activation, leading to attenuation of the GM-CSF mRNA binding capacity of all four AUF1 isoforms. This occurred despite the fact that only p40 and p45 contain Pin1 isomerization sites (Ser83-Pro84 and Ser87-Pro88). The resulting phenotype was enhanced GM-CSF mRNA stability and cytokine release. As GM-CSF blocks the default apoptosis of eosinophils, the excess release of prosurvival cytokine contributed to the accumulation of pathologic cells in the airways of active asthmatics. Conversely, Pin1 blockade prevented GM-CSF mRNA stabilization or cytokine secretion, and attenuated allergic inflammation and airway fibrosis in the rodent models of asthma [36]. Pin1 has since been implicated in the regulation of signaling mediated by parathyroid hormone and estrogen receptor as well [37,159]. In a rat model of secondary hyperparathyroidism, Pin1 interacted with phospho-KSRP, with isomerization contributing to its dephosphorylation. This increased the interaction between KSRP and PTH mRNA, resulting in reduced PTH mRNA stability and protein. Consistent with these results, the thyroid of Pin1 null mice displayed increased PTH mRNA levels and elevated serum PTH, indicating a possible role for Pin1 in the pathogenesis of hyperparathyroidism and related diseases. Moreover, in unstimulated tumor cells, the mRNA decay-promoting factor KSRP was required for the rapid degradation of β-catenin transcripts [82] whereas p38 MAPK-mediated phosphorylation of KSRP at a Pin1 site (pThr692-Pro693) impaired KSRP-RNA interactions and increased target mRNA abundance [81], suggesting that KSRP phosphorylation is also crucial for its mRNA binding capabilities (Table 1 and Figure 1). As AUF1, HuR, KSRP and TTP control the decay of many ARE containing mRNAs, Pin1 likely plays a broad role in the expression of additional cytokines, oncogenes and hormones in diverse cells and organs.

## 4. Effect of Pin1 Prolyl Directed, *Cis-Trans* Isomerization Activity on mRNA-AUBP Complex Remodeling

Post-translational modifications have impact on protein conformation and thus can alter the ability of AUBPs to bind ARE-containing transcripts. Depending on which AUBP occupies an ARE, the decay rate is increased or decreased. *In vitro* FRET experiments revealed that non-phosphorylated p40 AUF1 promotes formation of a condensed, less flexible structure within the TNF-α ARE [133]. While dually phosphorylated (Ser83 and Ser87) p40 AUF1 had a slightly lower binding affinity (two-fold) for the same ARE, it maintained the RNA in a less condensed and elongated form. As the phosphorylation of p40 at Ser83 and Ser87 was associated with stabilization of ARE transcripts, these data suggest that either loss of transcript contact as well as remodeling of protein interactions may underlie the changes in decay. We showed that suppression of phosphorylated AUF1 function during eosinophil or T cell activation requires isomerization by Pin1 [30,38]. In resting cells, catalytically inactive, phosphorylated Pin1 can bind to but not alter the ability of AUF1 to interact with and promote the degradation of GM-CSF mRNA. Leukocyte activation induced Pin1’s PPIase activity after PP2A mediated dephosphorylation [36], leading to a conformational transition in AUF1 that reduced its RNA-binding affinity. The mechanism for attenuated RNA binding may also include an inability to recruit critical cofactors including PM-Scl-75 [36] (Figure 1). The latter is particularly relevant as many protein cofactors have been identified as AUBP partners and include translation initiation factors, ubiquitinase, heat shock proteins, and nucleases [13]. Pin1-mediated ARE RNA metabolism is further supported by the observation that Pin1 is also associated with ARE-independent mRNA binding proteins such as SLBP and regulates PP2A-mediated mRNA decay and protein translation [26]. Pin1 regulated SLBP ubiquitination-modulated histone mRNA stability in a cell cycle-dependent manner. Phosphorylation of SLBP at Thr62 and Thr171 was crucial for the interaction with Pin1, which allowed dissociation of histone mRNA from SLBP. The Pin1-SLBP interaction was bipartite involving both the WW and PPIase domains [26,160], although the WW domain is known to have a 10-fold higher binding affinity for substrates than the PPIase domain [161,162]. In the majority of cases, both binding and catalytic activity of Pin1 are required for changes in the target protein molecular functions but in few circumstances, binding alone was reported to be sufficient [163,164,165]. Therefore, it is possible that in some situations Pin1 mediated prolyl *cis-trans* isomerization is not required for the effect of AUBPs on target mRNA turnover. Moreover, a role for phospho-HuR in modulating miRNA access to mRNA has been proposed by several independent studies, and may involve similar mechanisms as discussed above [166,167,168]. Collectively, modification-dependent AUBP regulation is similar to the recruitment of co-regulator complexes to gene promoters, and requires the simultaneous recognition of phosphoproteins and adjacent DNA sequences [169,170]. As Pin1 can bind transcription factors associated with many pro-inflammatory genes [22], Pin1 mediated prolyl *cis-trans* isomerization and phosphorylation-dependent changes in the architecture of AUBPs and local RNA/DNA structure provides a diverse population of molecular determinants to direct downstream macromolecular events.

## 5. Pin1 and Immune Disorders

ARE-mediated post-transcriptional regulation is particularly important for a rapid cellular response. This is observed during defense against pathogens, cardiovascular toning, organ rejection, and allergic reactions [22,140,171,172]. Primary dysregulation of Pin1 expression or its isomerase activity may be relevant for diseases where aberrant cell-cycle progression and cytokine production are contributing factors. This is likely the case in cancer where Pin1 is often overexpressed. Indeed, Pin1 overexpression has been linked to poor prognosis for breast cancer [22,163]. These data suggest that Pin1 could be an attractive therapeutic target. Given Pin1’s relationship to FKBP and cyclophilin families, we asked if Pin1 blockade could reduce the rejection of MHC mismatched lung transplants in rats. Splenic T cells from rats fed juglone, a moderately specific Pin1 inhibitor, showed significantly less IFN-γ and IL-2 mRNA and protein compared to untreated controls [84]. Pin1 KO T cells showed similar phenotypes to juglone treated, WT cells. IL-2 mRNA is stabilized by activated immune cells as a prelude to cytokine expression during allograft rejection. *In vivo* treatment with juglone not only suppressed cytokine production but also preserved transplanted organ architecture and function [84,158]. This observation suggests that Pin1 plays a role in mediating Type 1 immunity and organ rejection. Subsequent studies [36] also demonstrated an essential role for Pin1 in allergic disease. In animal models of asthma, systemic delivery of Pin1 inhibitors prevented airway inflammation by blocking the expression of eosinophil survival cytokines (GM-CSF and IL-5). T cells and eosinophils from juglone treated animals or Pin1 KO mice showed reduced cytokine mRNA and protein expression consistent with the post-transcriptional regulatory processes discussed above. In the context of primary viral infection, there is also growing evidence that Pin1 may be co-opted by viral proteins. For example, during virus infection, Pin1 isomerized viral integrase as well as cellular IRF3 required for efficient virus replication and IRF-3-dependent production of IFN-β, respectively [173,174]. As many immune disorders involve dysregulated IFNs (diabetes, multiple sclerosis and lupus), and GM-CSF production (macrophage mediated tissue damage, gastritis, myeloproliferative syndromes and eosinophilia) [158,175,176,177], Pin1 likely plays an important role in these pathologies through multiple mechanism controlling the production of immune mediators.

## 6. Conclusions

The regulation of mRNA stability through the ARE allows a fine-tuning of responses to extra- and intra-cellular signals. The process requires the regulated association of one or more AUBPs with target mRNAs in a sequential manner. Proper regulation of AUBPs is thus essential for normal cellular, tissue, and organ homeostasis. While kinase-mediated AUBP phosphorylation is critical, downstream events including Pin1 mediated binding and isomerization are also important. As seen in a variety of pathologies including cancer, allergy and infection, Pin1 dysregulation can have profound consequences. Critical, unanswered questions include the identification of additional Pin1 AUBP targets, characterization of how the *cis-trans* conversion alters AUBP binding to targets or cofactors and how drugs can be developed that can affect Pin1 or its interaction with protein ligands.
